# Supplementary material for: Phenolic Secondary Metabolites and Antiradical and Antibacterial Activities of Different Extracts of Usnea barbata (L.) Weber ex F.H.Wigg from Călimani Mountains, Romania
Source: Pharmaceuticals (Basel). 2022 Jul 4;15(7):829. doi: 10.3390/ph15070829 (PMC9322614; doi:10.3390/ph15070829)
Supplement: Supplementary file 1 [file pharmaceuticals-15-00829-s001.zip › pharmaceuticals-1781582-supplementary/Figures S1-S4 and Table S1.pdf]

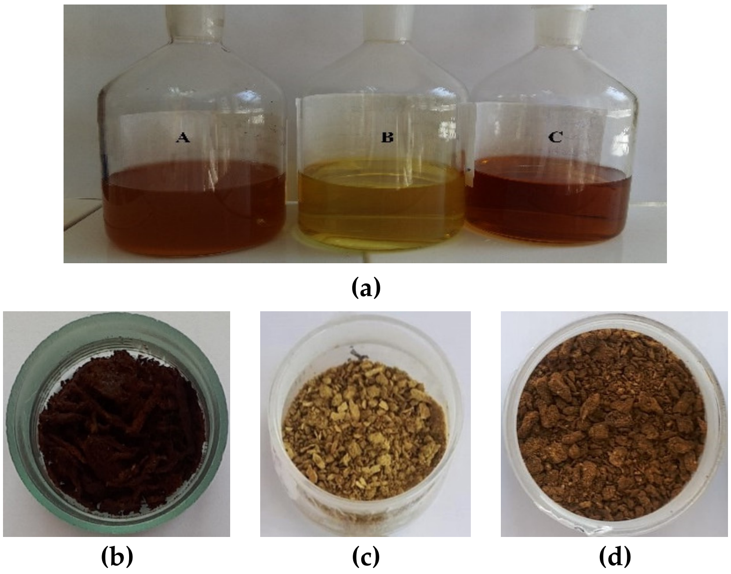

Figure S1. (a). *U. barbata* fluid extracts: A. *m*UBW, B. *m*UBA, C. *m*UBE; (b-d) *U. barbata* dry extracts: (b) *d*UBW, (c) *d*UBA, (d) *d*UBE.

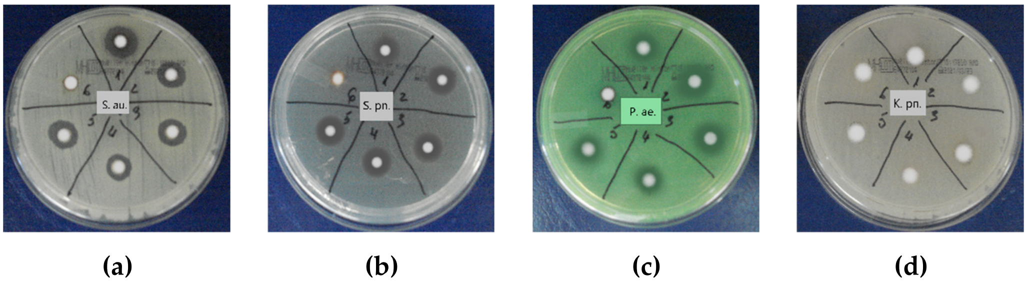

Figure S2. Antibacterial activity of usnic acid (1) and *U. barbata* extracts: *m*UBA (2), *d*UBA (3), *m*UBE (4), *d*UBE (5), *m*UBW (6), on *S. aureus* (a), *S. pneumoniae* (b), *P. aeruginosa* (c), *K. pneumoniae* (d).

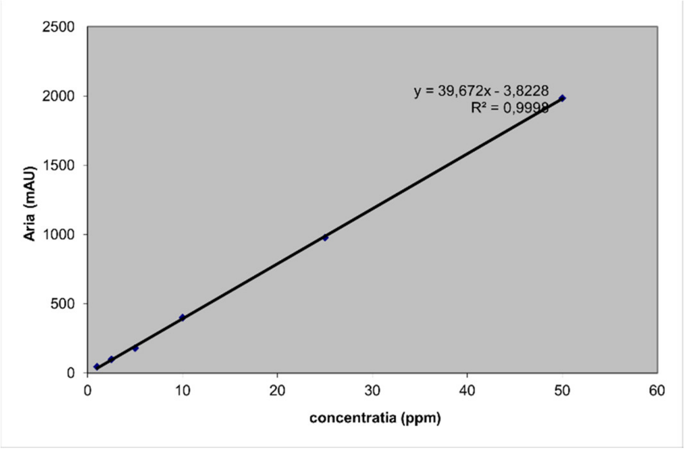

Figure S3. Calibration curve for usnic acid

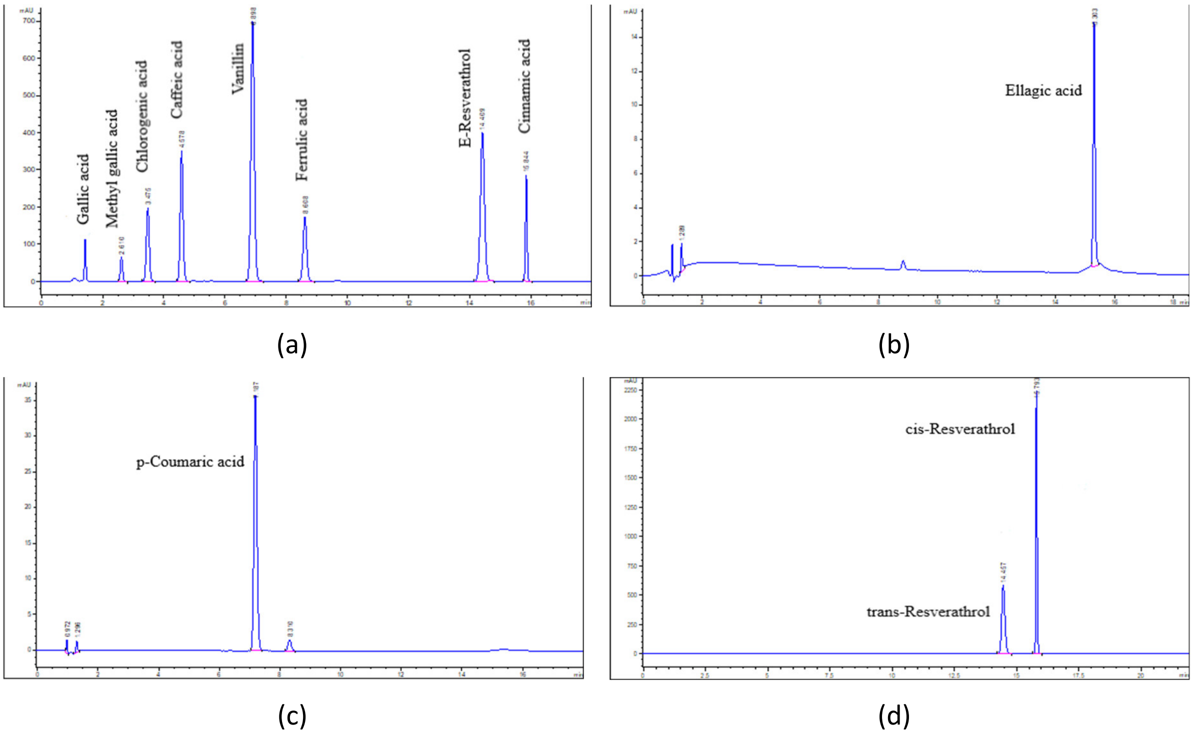

Figure S4. Polyphenols standards: mixture (a), ellagic acid (b), p-coumaric acid (c), cis-resveratrol and trans-resveratrol (d).

Table S1. Concentration, retention time, and correlation coefficient (R^2) values for all phenolic standards used in the HPLC-DAD method

Phenolic compound	Concentration (mg/mL)	Retention Time (min)	R^2
trans-Resverathrol (E)	0.22	14.467 ± 0.017	0.999
cis- Resveratrol (Z)	0.37	15.751 ± 0.058	0.997
Chlorogenic acid	0.37	3.501 ± 0.015	0.999
Caffeic acid	0.36	4.598 ± 0.036	0.996
Cinnamic acid	0.58	15.867 ± 0.007	0.998
Vanillin	0.42	6.919 ± 0.051	0.996
Gallic acid	0.39	0.990 ± 0.025	0.995
Ferrulic acid	0.38	8.565 ± 0.058	0.998
Ellagic acid	0.40	15.303 ± 0.027	0.998
p-Coumaric acid	0.41	7.187 ± 0.019	0.997
3 - Methyl gallic acid	0.51	2.606 ± 0.008	0.995
